# Pregnancy After *In Vitro* Fertilization in Budd–Chiari Syndrome Secondary to Polycythemia Vera: Multidisciplinary Management and Perioperative Considerations

**DOI:** 10.14740/jmc5319

**Published:** 2026-05-25

**Authors:** Nikolina Musulin, Sonja Krofak, Aleksandra Jokic, Ivana Petra Kolarek, Milan Pavlovic, Ivanka Bekavac Vlatkovic

**Affiliations:** a Department of Obstetrics and Gynecology, KB Sveti Duh, 10000 Zagreb, Croatia; b Clinic for Anesthesiology, Reanimatology and Intensive Care, KB Sveti Duh, 10000 Zagreb, Croatia; c Faculty of Kinesiology, University of Zagreb, 10110 Zagreb, Croatia

**Keywords:** Budd–Chiari syndrome, *In vitro* fertilization, Portal hypertension, Terlipressin, Intrahepatic cholestasis of pregnancy

## Abstract

Budd–Chiari syndrome (BCS) is a rare hepatic vascular disorder frequently associated with prothrombotic conditions such as myeloproliferative neoplasms. Pregnancy and assisted reproductive technologies further increase thrombotic and portal-hypertensive risk. We report a successful *in vitro* fertilization–conceived pregnancy in a 35-year-old woman with chronic BCS secondary to JAK2-positive polycythemia vera. The pregnancy was complicated by ovarian hyperstimulation syndrome and severe intrahepatic cholestasis of pregnancy. Progressive portal hypertension with worsening esophageal varices prompted planned preterm delivery at 34 weeks’ gestation. A multidisciplinary team performed cesarean delivery under epidural anesthesia with prophylactic perioperative terlipressin administration and thromboprophylaxis. Maternal and neonatal outcomes were favorable. With careful multidisciplinary management, pregnancy in women with stable BCS is feasible.

## Introduction

Budd–Chiari syndrome (BCS) is defined as hepatic venous outflow obstruction located anywhere from the small hepatic venules to the junction of the inferior vena cava and the right atrium [1]. Most cases are thrombotic and occur in the setting of an underlying prothrombotic condition such as myeloproliferative neoplasms, inherited thrombophilia, or antiphospholipid syndrome [1, 2].

Diagnosis is imaging-based, and treatment follows a stepwise approach, with long-term anticoagulation as the cornerstone of therapy [2]. Pregnancy adds hemostatic and hemodynamic stressors and increases maternal complications such as ascites and variceal bleeding [3].

Contemporary cohort studies indicate that pregnancy can be successful in women with stable, treated BCS when managed within a multidisciplinary setting [4–6].

We report the management of a pregnancy conceived via *in vitro* fertilization (IVF) in a woman with chronic BCS secondary to JAK2-positive polycythemia vera (PV). The novelty of this case lies in the successful assisted conception and the multidisciplinary management of a complex pregnancy complicated by BCS and PV.

## Case Report

### Investigations

A 35-year-old woman with chronic BCS was diagnosed at 25 years of age after developing portal vein thrombosis shortly after beginning combined oral contraceptives. Workup confirmed an underlying myeloproliferative neoplasm, PV (JAK2-positive). The disease remained clinically stable for 10 years on hydroxyurea and warfarin.

Following preconception counseling, pregnancy was achieved through an IVF protocol. Warfarin was replaced with low-molecular-weight heparin (LMWH) at a dose of 5,000 IU subcutaneously. Prophylactic dosing was selected due to the increased bleeding risk associated with portal hypertension and varices. Hydroxyurea was discontinued because of teratogenicity approximately 18 months before conception as part of preconception counseling, although conception occurred later during this period.

Anti–factor Xa activity remained < 0.1 IU/mL during LMWH therapy.

Early pregnancy was complicated by ovarian hyperstimulation syndrome (OHSS), with clinically evident abdominal distension due to ascites. First-trimester ultrasound confirmed occlusion of all three hepatic veins with features of mild portal hypertension.

The patient had been undergoing surveillance for esophageal varices that had been treated with endoscopic band ligation prior to pregnancy. At 29 weeks’ gestation, pregnancy-associated hypervolemia likely contributed to increased portal pressure and progression of esophageal varices, which were non-collapsible on air insufflation.

Non-selective beta-blocker therapy for variceal bleeding prophylaxis was considered. Although carvedilol is currently preferred, the patient had previously experienced hypotension and fatigue while using propranolol. Due to prior intolerance and concerns regarding hemodynamic stability during pregnancy, beta-blocker therapy was deferred.

### Diagnosis

At 28 weeks’ gestation, the patient developed generalized pruritus accompanied by elevated total bile acids (29.8 µmol/L), consistent with intrahepatic cholestasis of pregnancy (ICP). Treatment with ursodeoxycholic acid (UDCA) was initiated at 250 mg three times daily and subsequently escalated to 500 mg three times daily in response to persistently elevated bile acid concentrations. A course of antenatal corticosteroids was administered in anticipation of potential preterm delivery.

Rifampicin was considered but deferred because of potential hepatotoxicity and bleeding risk.

Despite therapy, serial laboratory assessments demonstrated a progressive rise in bile acid concentrations, reaching a peak of 101.8 µmol/L in the third trimester. Concurrent laboratory findings included anemia (hemoglobin 96–108 g/L) and thrombocytopenia (platelet count 100–152 × 10^9^/L). Total bilirubin ranged from 22 to 45 µmol/L. Serial biochemical liver function tests were performed several times per week under gastroenterology supervision. Results showed aspartate aminotransferase (AST) 21–34 IU/L, alanine aminotransferase (ALT) 13–30 IU/L, gamma-glutamyl transferase (GGT) 25–33 IU/L, alkaline phosphatase (ALP) 163–192 IU/L (stably elevated throughout pregnancy, consistent with pregnancy-related or placental ALP fraction), total bilirubin 22–45 µmol/L, albumin 32–39 g/L, plasma ammonia 31–62 µmol/L, and prothrombin time/international normalized ratio (INR) (PV 77–100%, INR 1.03–1.26). These findings indicated preserved hepatic synthetic function with mild cholestatic changes consistent with ICP.

Fetal growth and surveillance were reassuring.

### Treatment (perioperative and postoperative management)

Delivery planning involved maternal–fetal medicine, hepatology, hematology, anesthesiology, and neonatology specialists.

At 34 weeks’ gestation, preterm delivery was recommended due to worsening portal hypertension and severe cholestasis. A strategy including prophylactic perioperative terlipressin was selected to reduce portal-hypertensive risk.

Cesarean section was elected due to the patient’s underlying portal-hypertensive cirrhosis with previously ligated but progressive esophageal varices, in order to minimize the risk of bleeding associated with increased intra-abdominal pressure during vaginal delivery. The presence of severe ICP further supported the decision for planned preterm cesarean delivery under controlled multidisciplinary conditions.

Cesarean section under epidural anesthesia resulted in delivery of a preterm male neonate, with a birth weight of 2,020 g and length of 46 cm. Apgar scores were 10 at 1 and 5 min. Estimated blood loss was approximately 500 mL. Terlipressin was administered as a single perioperative 1 mg intravenous dose, followed by 1 mg intravenously every 6 h during the first 24 postoperative hours, in combination with albumin, diuretics, antibiotic prophylaxis, and thromboprophylaxis in the obstetrics and gynecology intensive care unit. Laboratory evaluation demonstrated elevated ammonia levels (87 µmol/L) and hyperbilirubinemia in the absence of clinical signs of hepatic encephalopathy, prompting initiation of lactulose therapy. Large-volume ascites was drained; cytology was unremarkable. The patient remained hemodynamically stable without infectious, neurological, or cardiopulmonary complications.

### Follow-up and outcomes

The puerperium was uncomplicated. Postpartum magnetic resonance imaging (MRI) demonstrated persistent hepatic vein occlusion with established cirrhotic changes. Laboratory evaluation showed an elevated alpha-fetoprotein level (AFP) (41.17 µg/L), and the patient’s Model for End-Stage Liver Disease (MELD) score was 15. Given these findings, she was referred to a tertiary hepatology center for ongoing evaluation, including repeat AFP testing and long-term surveillance for portal-hypertensive and hepatocellular carcinoma–related complications, and planning of further management or potential interventional strategies.

She was also reviewed by a hematologist at our institution, who recommended continuation of low-molecular-weight heparin (LMWH) for 6 weeks postpartum, with subsequent reassessment for possible transition to oral anticoagulation.

Follow-up data beyond the referral are not available from our institution. At the time of discharge and early follow-up, the patient remained clinically stable.

A summary of the clinical timeline is presented in Table 1.

**Table 1:** Chronological Clinical Course Summarizing Key Events, Findings, and Management Decisions From Pre-Conception to Postpartum

Time period/gestational age	Clinical event	Key findings	Management/interventions
Pre-conception period	Known Budd–Chiari syndrome and polycythemia vera	Chronic hepatic venous outflow obstruction	Long-term anticoagulation; hepatology and hematology follow-up
Early pregnancy (post-IVF conception)	IVF pregnancy complicated by OHSS	Ascites	LMWH; monitoring
Third trimester	Progression to severe ICP and portal hypertension; Decision for planned early delivery	Elevated bile acids; rising bilirubin; thrombocytopenia	UDCA therapy; Intensified surveillance; multidisciplinary decision for preterm cesarean section
Delivery	Preterm cesarean section	High maternal risk due to BCS, PV, severe ICP, portal hypertension	Perioperative terlipressin; regional anesthesia; close monitoring
Immediate postpartum period	Uncomplicated recovery	No hemorrhagic or thrombotic events	Continuation of LMWH; standard postpartum care
Postpartum follow-up	Long-term hepatic evaluation	MRI: cirrhotic-pattern changes; MELD 15; mildly elevated AFP	Hepatology follow-up; ongoing surveillance

AFP: alpha-fetoprotein; BCS: Budd–Chiari syndrome; ICP: intrahepatic cholestasis of pregnancy; IVF: *in vitro* fertilization; LMWH: low-molecular-weight heparin; MELD: Model for End-Stage Liver Disease; MRI: magnetic resonance imaging; OHSS: ovarian hyperstimulation syndrome; PV: polycythemia vera; UDCA: ursodeoxycholic acid.

## Discussion

BCS is a chronic condition in which long-standing obstruction of hepatic venous outflow may lead to progressive portal hypertension and, in some cases, cirrhosis. Despite advances in diagnosis and therapy, long-term anticoagulation remains the foundation of management, with escalation to endovascular or surgical interventions when clinically indicated [1, 2].

Pregnancy in women with BCS requires individualized risk assessment with careful attention to thromboprophylaxis, hemodynamic stability, and monitoring of portal-hypertensive complications. Published series report high live-birth rates with LMWH but consistently higher rates of maternal morbidity compared with the general obstetric population [4–6]. In our patient, concomitant PV further amplified baseline thrombotic risk and necessitated tailored hematologic evaluation and anticoagulant management [7, 8].

The fact that pregnancy was achieved via assisted reproduction significantly influenced the clinical course. OHSS is a well-recognized prothrombotic state that can induce marked hemodynamic shifts in early pregnancy [9]. Our patient developed severe intrahepatic cholestasis following an episode of OHSS. Recent case-based literature and systematic reviews increasingly describe early-onset or severe cholestasis in pregnancies conceived through IVF and complicated by OHSS, supporting heightened clinical vigilance in comparable scenarios [10].

Portal-hypertensive liver disease during pregnancy is inherently high risk and requires close collaboration among hepatology, maternal–fetal medicine, hematology, gastroenterology, and anesthesia teams. Management must balance the competing risks of hemorrhage and thrombosis, particularly in women with pre-existing venous outflow obstruction [3, 11, 12].

The use of terlipressin presents an additional therapeutic challenge in pregnancy. Although widely employed for acute variceal hemorrhage in non-pregnant adults [13], its application during pregnancy remains controversial because of concerns regarding uterine vasoconstriction and reduced uteroplacental perfusion. Nevertheless, terlipressin has been described in obstetric and gynecologic emergencies when standard measures failed, including intramyometrial use for refractory atonic postpartum hemorrhage and peripartum hemostasis in miscarriage-related bleeding [14]. These isolated reports suggest that, in highly selected and life-threatening situations, short-term terlipressin exposure may offer meaningful hemostatic benefit, although evidence remains limited and off-label.

### Learning points

In our case, prophylactic perioperative terlipressin was administered because of the anticipated high-risk combination of portal hypertension, cytopenias, and severe cholestasis at preterm delivery, under close intensive care monitoring. No hemorrhagic or thrombotic complications were observed; however, a single case cannot establish safety. More broadly, pregnancy can be successfully achieved in women with stable BCS when managed in a tertiary multidisciplinary setting. Assisted reproduction and ovarian hyperstimulation may further increase thrombotic and portal-hypertensive risks. Severe ICP may precipitate hepatic decompensation and necessitate earlier delivery, underscoring the importance of coordinated peripartum planning to balance bleeding and thrombotic complications.

### Conclusion

Pregnancy in stable, treated BCS is high risk but can be successful with multidisciplinary management. Recognition of IVF-related complications and individualized delivery planning are essential. Terlipressin use should remain limited to exceptional situations.

## Data Availability

The authors declare that data supporting the findings of this study are available within the article.

## Abbreviations

AFP: alpha-fetoprotein
BCS: Budd–Chiari syndrome
ICP: intrahepatic cholestasis of pregnancy
IVF: *in vitro* fertilization
LMWH: low-molecular-weight heparin
MELD: Model for End-Stage Liver Disease
MRI: magnetic resonance imaging
OHSS: ovarian hyperstimulation syndrome
PV: polycythemia vera
UDCA: ursodeoxycholic acid
